# Comparative Lipidomics of Different Yeast Species Associated to *Drosophila suzukii*

**DOI:** 10.3390/metabo10090352

**Published:** 2020-08-28

**Authors:** Flavia Bianchi, Urban Spitaler, Peter Robatscher, Rudi F. Vogel, Silvia Schmidt, Daniela Eisenstecken

**Affiliations:** 1 Laboratory for Flavours and Metabolites, Institute for Agricultural Chemistry and Food Quality, Laimburg Research Centre, Ora (BZ), 39040 Auer, Italy; flavia.bianchi@laimburg.it (F.B.); peter.robatscher@laimburg.it (P.R.); 2 Chair of Technical Microbiology, School of Life Sciences Weihenstephan, Technical University of Munich, Gregor-Mendel-Straße 4, 85354 Freising, Germany; rudi.vogel@wzw.tum.de; 3 Entomology Group, Institute for Plant Health, Laimburg Research Centre, Ora (BZ), 39040 Auer, Italy; urban.spitaler@laimburg.it (U.S.); silvia.schmidt@laimburg.it (S.S.); 4 Institute of Plant Protection, Department of Crop Sciences, University of Natural Resources and Life Sciences, Gregor-Mendel-Straße 33, 1180 Vienna, Austria

**Keywords:** lipidomic profile, yeast strains, spotted wing drosophila, untargeted LC-MS

## Abstract

Yeasts constitute a dietary source for the spotted wing drosophila (SWD) and produce compounds that attract these flies. The study of the chemical composition of the yeast communities associated with SWD should therefore help to understand the relationship between the biology of the insect and the yeast’s metabolism. In the present study, the lipidome of five yeast species isolated from grapes infested by SWD (three *Hanseniaspora uvarum* strains, *Candida* sp., *Issatchenkia terricola*, *Metschnikowia pulcherrima* and *Saccharomycopsis vini*) and a laboratory strain of *Saccharomyces cerevisiae* was explored using an untargeted approach. Additionally, the lipid profile of two species, *S. cerevisiae* and *H. uvarum*, which were reported to elicit different responses on SWD flies based on feeding and behavioral trials, was compared with a chemical enrichment approach. Overall, 171 lipids were annotated. The yeast species could be distinguished from each other based on their lipid profile, except for the three strains of *H. uvarum,* which were very similar to each other. The chemical enrichment analysis emphasized diversities between *S. cerevisiae* and *H. uvarum*, that could not be detected based on their global lipid profile. The information concerning differences between species in their lipidome may be of interest to future entomological studies concerning the yeast-insect interaction and could help to explain the responses of SWD to diverse yeast species.

## 1. Introduction

*Drosophila suzukii* Matsumura, the spotted wing drosophila (SWD), is an insect pest which causes severe economic losses and agricultural damages in Italy as well as in other countries worldwide [1,2,3,4]. Different yeast species present on fruits are found in association with SWD [5,6,7], and due to their attractiveness and feeding stimulant activity towards SWD flies, these microorganisms can be employed in baits or attract-and-kill formulations against SWD [8,9,10,11]. In fact, yeasts constitute a food source for *Drosophila* flies [12] as they provide protein, amino acids, lipids, and vitamins to the insect [13,14,15,16,17]. In addition, yeasts can stimulate appetite behavior in SWD flies [7] and encourage insects to feed on yeast-laden food, because they associate it with a sugar source [18]. Yeasts also produce volatile compounds that are attractive to SWD flies [5,9,19,20]. Not only the yeast species, but also the strain and growth medium affect the attractiveness [20].

The nutritional value of a food source for *Drosophila melanogaster* is influenced by the protein-to-carbohydrates-ratio [21], and vitamins provided by symbiotic microorganisms are essential food components in the *Drosophila* diet [12,22]. The same 10 amino acids which are essential to other eukaryotes are also necessary for *Drosophila* [23], but the requirement of other compounds like proteins, carbohydrates, and lipids are different among *Drosophila* species, and their availability influences the fitness and life-history traits of the insect [24,25].

Although *Drosophila* is able to synthetize the necessary fatty acids for its survival [14], little is known about the preferences and nutritional behavior of *Drosophila* fed with different lipid-containing diets. Carvalho et al. [14] demonstrated that the dietary fatty acids’ composition has an effect on the phospholipid compounds present in cell membranes of *Drosophila melanogaster*. In contrast, the abundance of specific phospholipid classes in various tissues does not reflect the phospholipidic composition of their diet [14]. Lipids are ubiquitous compounds with a key role in numerous biological processes: they are (i) constituents of cell membranes, (ii) involved in signaling pathways, and (iii) a storage energy source [26]. The lipid composition of eukaryotic organisms is influenced by the carbon source and the growth medium composition [27,28]. Therefore, differences in the lipid profile are reflected in the phenotype in specific environmental conditions [29]. Yeasts constitute a lipid source for *Drosophila* and their lipidome is representative for numerous compound classes. Triacylglycerols (TG) and steryl esters (SE) are reported as the two major nonpolar lipids of the yeast *S. cerevisiae* [30]; besides representing an energy source, they also constitute building blocks for lipid membranes [30] and are important for controlling the cellular levels of fatty acids and sterols [31]. Other important yeast lipid classes include sphingolipids, which are involved in the regulation of numerous cellular processes [32]; unsaturated and saturated fatty acids (FA), which are essential constituents of biomembranes [33]; phospholipids [34,35], and sterols, including ergosterol, which contributes to membrane integrity and is reported as the major sterol in *S. cerevisiae* [36]. The production of volatile compounds by yeasts is influenced by their lipid profile and the availability of lipid sources in the yeast growth medium [37,38], with differences among strains [38] and species [39]. In addition, free FAs are precursors of volatile compounds such as esters, alcohols, and aldehydes [39,40,41].

Lipidomics is an innovative tool that can be used to understand the biological role of lipids. Despite the complexity of the lipidome of eukaryotic cells, the progress in high throughput techniques based on mass spectrometric approaches allowed the exploration of numerous compound classes. This provides new insight into the molecules and the molecular pathways involved in lipid metabolism. The study of the cellular lipidome involves the description of the functions of lipids and therefore requires knowledge about the molecular basis of the differences in the lipid profiles of certain organelles, the interaction and signaling pathways that involve lipids, and the regulation of the local concentration of lipids in cell compartments [42]. The large number of different existing molecular classes requires complex sample preparation procedures and computational and bioinformatic interpretation of the data, which constitute some of the limitations that may arise in lipidomic studies [43]. Thanks to its relatively simple lipidome and the knowledge of the function and regulation of genes involved in its lipid metabolism, yeasts offer numerous advantages for such studies [44].

Despite the high number of scientific publications focused on lipidomics [35,43,45,46,47,48], few studies are available concerning comparative yeast lipidomics [29,49], including publications from the 1970s to the 1990s [34,50,51], and none of these are focused on yeast-insect associations.

In this study, for the first time, the lipidomes of different yeast species associated to SWD were compared. Untargeted lipidomics based on reversed-phase liquid chromatography–quadrupole/time-of-flight mass spectrometry (RPLC-QTOFMS) was performed on yeast extracts. Six species (*H. uvarum*, *S. cerevisiae*, *Candida* sp., *I. terricola*, *M. pulcherrima* and *S. vini*) were chosen based on previous studies [52]. *H. uvarum* had a beneficial effect on the survival of SWD larvae [6]. When given as a food source to SWD females, this yeast positively influenced their fecundity; while in capillary feeding assays this species was found to increase ingestion and decrease mortality of SWD adults compared to other yeasts [52]. It was found to be more attractive [19] and to be a preferable food source compared to other yeasts [7]. Therefore, three strains (H. u. 1.23, H. u. 2.2 and H. u. 3.4) of this species were included in the study. *S. cerevisiae* was included as a reference yeast, since detailed information concerning the genome, protein data, and lipidome of this species are available [29,35,53]. The other selected species have been already isolated from grapes infested by SWD [6], with *H. uvarum* being frequently found in association with SWD [10,54] and reported as one of the predominant species [5,55].

The description of the profile of non-polar metabolites of yeasts provides insight about the metabolism of different species cultivated in the same conditions, as well as a list of potential compounds, which may be relevant for the ecology of SWD, and therefore involved in the interaction between microorganisms and SWD.

## 2. Results and Discussion

### 2.1. Compound Annotation and Differences in the Lipid Profiles

The lipidome of the eight selected yeasts was explored. Six cultures of each yeast were included in the dataset to take into account the biological variability. To ensure that all biological replicates were in the same metabolic conditions, all yeast cultures were cultivated under the same growth conditions, and samples were collected at the same time upon reaching the stationary growth phase.

Overall, 171 compounds, including phospholipids (GP), sterols, fatty acids (FA), ceramides (Cer), sphingoid long-chain bases (LCB), monoglycerides (MG), diglycerides (DG) and triglycerides (TG) were annotated. Only known compounds were considered since this study was aimed at finding compounds potentially involved in the interaction between yeasts and SWD. Retention time, ionization mode, annotation level, and mass error (ppm) are reported for each of the annotated compounds in the Supplementary Materials (Table S1). Based on the results of the analysis of variance (ANOVA), significant differences (*p* = 0.05) were found among yeasts concerning all annotated compounds, except for FA(18:0) and DG(16:0_18:1).

**Figure 1:**
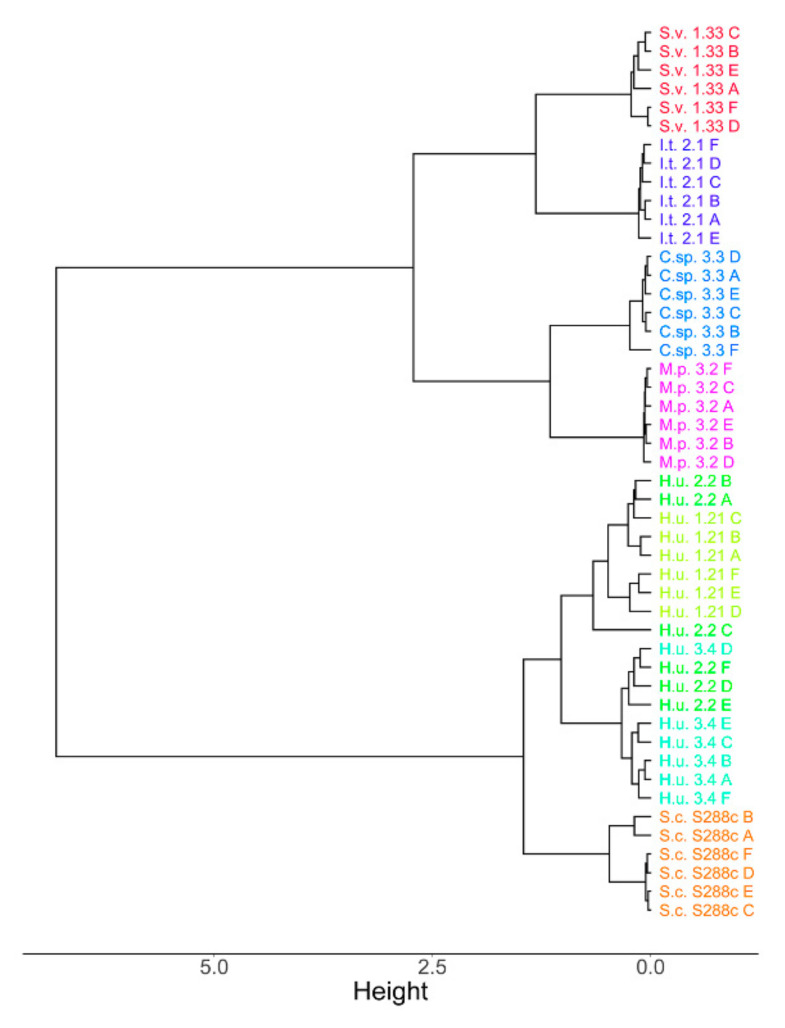
Hierarchical clustering dendrogram obtained using Spearman distance and the Ward clustering algorithm including all the annotated metabolites for each of the six biological replicates (A to F) per yeast.

A hierarchical clustering dendrogram was generated using Spearman distance and the Ward clustering algorithm to visualize the dataset. The clustering results show that the biological variability is lower in comparison to the differences among species since the species can be divided into subgroups (Figure 1). Two main clusters were observed: cluster one, including C.sp. 3.3, M.p. 3.2, S.v. 1.33 and I.t. 2.1, and cluster two, including the three *H. uvarum* strains plus S.c. S288c, indicating some similarities in the profiles of lipids in these two groups of species. The three strains belonging to *H. uvarum* clustered together, indicating a strong similarity between them. Except for S.c. S288c and I.t. 2.1, both of which belong to the family *Saccharomycetaceae* [56,57,58], the investigated yeasts belong to different families; therefore, the clustering found does not appear to reflect taxonomic relationships, though specific taxonomic tools and taxonomically defined strains should be considered for classification purposes. This is not surprising, since changes in the lipid metabolism can easily occur [44] and are influenced by the growth conditions and compounds present in the growth medium [27,28], which can supposedly differentially affect the lipid metabolism in the various yeast species.

### 2.2. Compound Classes Responsible for Discrimination between Yeast Species

**Figure 2:**
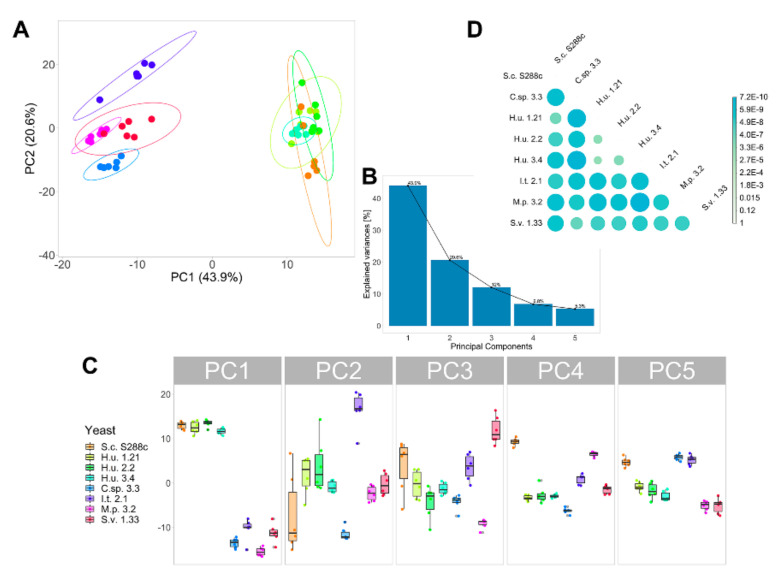
(**A**) Two-dimensional score plot generated using the first two principal components of the PCA. PCA was performed including all the 171 compounds annotated in six yeasts under investigation. (**B**) The scree plot shows the variance explained by the first five principal components. (**C**) Box plots of the scores of each yeast under investigation for the first five principal components. (**D**) The result of the pairwise MANOVA tests between yeast species based on the first five principal components of the PCA is reported as a heat map indicating significant differences in the yeast lipid chemistry.

A principal component analysis (PCA) was performed to highlight the differences between the six yeast species or strains (Figure 2A). Based on the 171 metabolites found, the yeast species could be sorted into discrete clusters. The three strains of *H. uvarum* clustered together. Augustyn et al. [51] reported an absence of variability among different species in the genus *Hanseniaspora* based on the cellular FA profile. The results of this study confirm the low variation among strains of this species, also extending the comparison to other lipid classes. The first two principal components explained 64.5% of the variation in the lipid profile, with principal component one accounting for 43.9% and principal component two for 20.6% of the total variation (Figure 2A,B). GPs, DGs, and TGs containing polyunsaturated fatty acids (PUFAs) negatively influenced the first principal component as well as unsaturated free FAs and ergosterol. Principal component two was strongly influenced in a positive direction by most of the FAs, including PUFAs, and negatively by most of the GPs. The statistical significance of the differences among the overall yeast lipidome profiles was determined by pairwise multivariate analysis of variance (MANOVA) of the first five principal components. Principal components four and five were included for the calculation of the MANOVA since they were found to be more informative for the discrimination between *H. uvarum* strains and *S. cerevisiae* compared to the first three principal components (Figure 2C). Except for H.u. 2.2 and H.u. 1.21 (*p* = 0.011), H.u. 1.21 and H.u. 3.4 (0.026), all the species are significantly different from each other (*p* = 0.005) (Figure 2D).

### 2.3. Differences in the Lipid Profile of GP, DG and TG

**Figure 3:**
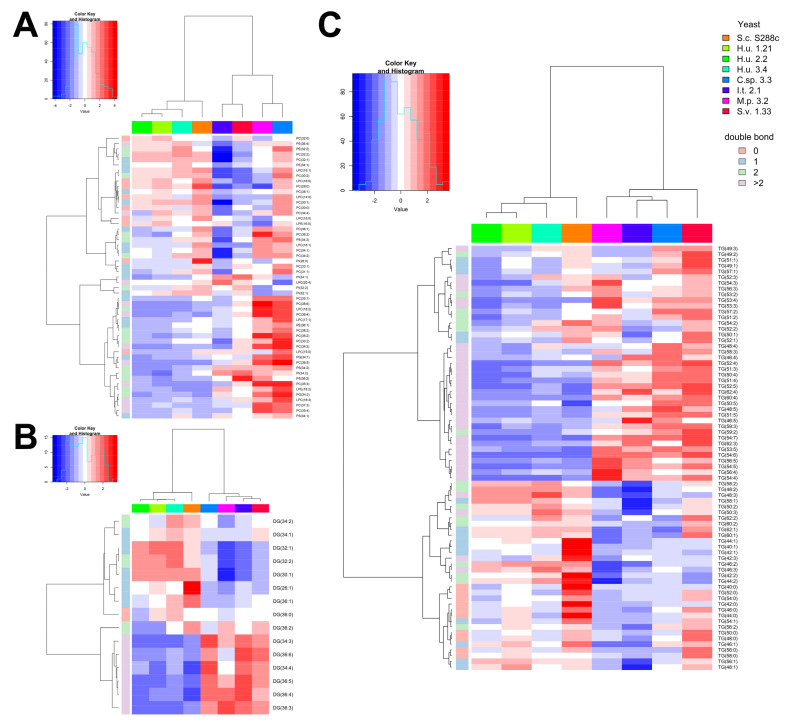
Heatmaps of GPs (**A**), DGs (**B**), and TGs (**C**) in the yeast species and strains included in the study. Intensities of single compounds are displayed using a color scale ranging from red (higher values) to blue (lower values), as shown in the legend. Both rows and columns are clustered using Spearman distance and a Ward clustering algorithm. Average values (n = 6) for each yeast are shown.

Different classes of phospholipids were found in the analyzed yeast samples, including phosphatidylserine (PS), phosphatidylcholine (PC), phosphatidylethanolamine (PE), phosphatidylglycerol (PG), phosphatidylinositol (PI), lysophosphatidylethanolamine (LPE) and lysophosphatidylcholine (LPC). To visualize the contribution of DGs and TGs as well as the one of GPs to the discrimination between species, heatmaps for different compound classes are shown separately (Figure 3). A similar GP, DG, and TG pattern between the three *H. uvarum* strains was observed. According to the findings of Hein and Hayen [29], the GP pattern was found to be useful to discriminate between phylogenetically different yeast species. Two species, C.sp. 3.3 and M.p. 3.2, were generally richer in GPs compared to the other analyzed (Figure 3A). In agreement with the findings of Hein and Hayen [29], S.c. S288c was found poor in GPs containing fatty acids with more than two double bonds. Previous studies confirm the lack of PUFAs in *S. cerevisiae* [49], which is reflected in the presence of only saturated and monounsaturated FAs in the acyl chains of GPs. The same could be observed in the three *H. uvarum* strains, in accordance with previous studies [51] that reported the absence of C18:2, C18:3, and C16:2 FA both in *S. cerevisiae* and *H. uvarum*. TGs were found informative for discrimination between the two species *H. uvarum* and *S. cerevisiae* since their profiles are not similar (Figure 3C). Considering DGs and TGs, the main difference between the two big clusters from the hierarchical clustering analysis, namely cluster one including C.sp 3.3, M.p 3.2, I.t. 2.1, S.v. 1.33 and cluster two comprising *H. uvarum* and S.c. S288c, is the lower amount of compounds containing PUFAs in cluster two. S.v. 1.33 was generally found to possess higher concentrations of a larger variety of DGs and TGs compared to the other yeasts. Interestingly, the presence of odd-numbered fatty acids in the acyl chains of TGs, PCs and LPCs was found in the three species C.sp 3.3, M.p 3.2, and S.v. 1.33. In particular, S.v. 1.33 contained the highest amounts in TGs containing odd-numbered fatty acids, while higher amounts of PCs and LPCs with this characteristic were found in C.sp 3.3. Previous studies indicated that odd-chain fatty acids in yeasts are produced from the elongation of an odd-chain precursor rather than *de novo* synthesis [59], though in some *Candida* species a small amount of odd-numbered fatty acids was observed even in cells cultivated on even-numbered alkanes [60]. Since microbial lipids mainly contain even-numbered FAs [61,62], this peculiarity constitutes an additional parameter to be considered for discrimination between species.

### 2.4. Differences in the Lipid Profile of FA, Ceramides, LCB and Sterols

**Figure 4:**
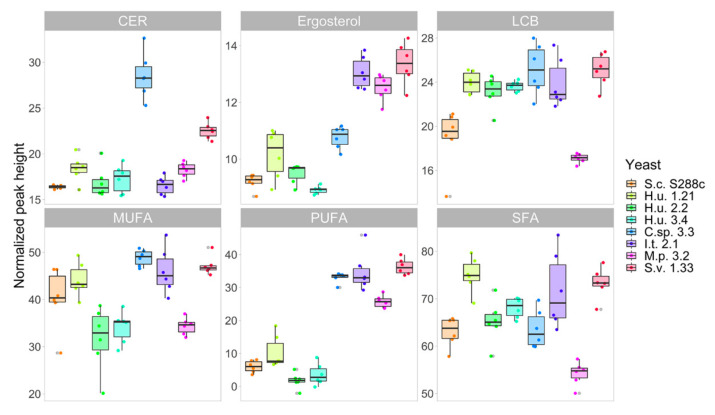
Boxplot of total ceramides (Cer), ergosterol, long-chain base (LCB), monounsaturated fatty acids (MUFA), polyunsaturated fatty acids (PUFA), and saturated fatty acids (SFA) in the eight yeasts included in the study (n = 6).

Kim et al. [63] showed that the gene Gr64e controls the behavioral as well as the electrophysiological responses of *Drosophila* to fatty acids, indicating that these compounds are detected by the insect’s gustatory system. In capillary feeding assays, *Drosophila* flies were found to prefer a fatty acid solution rather than water [64]. According to these data, the differences in the chemical compositions of fatty acids among yeast species associated with SWD were of great interest to this study. Oleic acid, palmitoleic acid, palmitic acid, and stearic acid are reported as the major FA of *S. cerevisiae* [27,36,50]. Viljoen et al. [50] also state that the *Saccharomycetaceae* family, to which *S. cerevisiae* belongs, is characterized by a higher concentration of oleic acid compared to the *Saccharomycodaceae*, the family to which *H. uvarum* belongs, and the *Metschnikowiaceae*, the family of *M. pulcherrima.* A peculiarity of *S. cerevisiae* is that it is unable to produce PUFAs with more than two double bonds, while other species can produce unsaturated FAs with a variable number of double bonds [65]. More than 20 different FAs were found in the analyzed samples, with differences among species (Table S1). Results confirm the lack of PUFAs in *S. cerevisiae* (Figure 4), while S.v. 1.33 was the richest species in oleic acid, one of the fatty acids investigated in the above-mentioned studies [63,64]. The three *H. uvarum* strains show, similarly to *S. cerevisiae*, low amounts of PUFAs (Figure 4). It should be noted that very-long-chain fatty acids with chains longer than 20 carbon atoms were also annotated. These compounds are important lipid components found in all organisms, including *S. cerevisiae* [66] and, due to the high variability of their concentrations, as well as the complexity of their detection and identification without employing LC-MS techniques [61], only a few studies are dedicated to this group of compounds. Ceramides and long-chain bases (LCB) are constituents of sphingolipids. Four different ceramides and the two LCB phytosphingosine (PHS) and dihydroshphingosine (DHS) were found in the samples analyzed. Considering sterols, only ergosterol was found, with higher relative amounts in S.v. 1.33, I.t. 3.2, and M. p. 3.2 and the lowest amount in H.u. 3.4 (Figure 4).

### 2.5. Relationship between the Selected Yeasts and SWD

**Figure 5:**
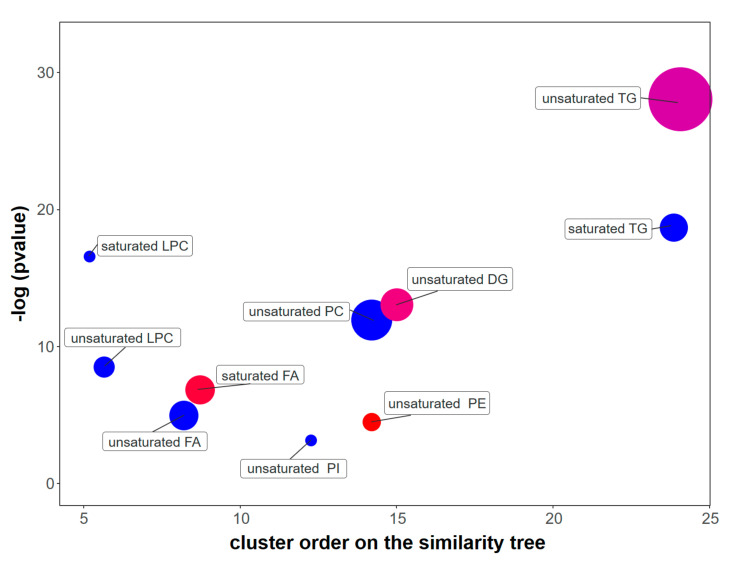
ChemRICH results plot shows the comparison between H.u. 3.4 and S.c. S288c. Each node represents a significantly altered cluster of lipids. The most significantly impacted lipid clusters are at the top of the *y*-axis. Node sizes account for the number of lipids in the clusters, while the node color scale represents the proportion of lipids having higher (red) or lower (blue) concentrations in H.u. 3.4 compared to S.c. S288c. The purple node indicates that in the clusters of unsaturated DG and TG, there is a number of compounds that are more concentrated in H.u. 3.4, and other compounds that are more abundant in S.c. S288c. Only significantly impacted clusters are shown (*p* = 0.05).

The variety and diversity of yeast species associated to SWD as well as the wide range of host fruits reflect the insect’s ability to adapt to nutrient sources available in the environment [67]. Feeding preferences of *Drosophila* flies for different yeast-based diets are influenced by numerous factors, including the volatile compounds emitted by yeasts as well as the nutritional composition of the food source [9,12]. Yeasts influence several life-history traits of *D. melanogaster* with different effects on larvae and adult flies [68]. This complex interaction mechanism between yeasts and *Drosophila* has been widely studied from a behavioral and ecological point of view [19,25] and through the exploration of the volatile compounds involved in attractiveness mechanisms [12,19]. However, little is known about the role of yeast specific metabolites in the association with the insect. In light of previous studies, the results of the present work were evaluated in relation to SWD’s dietary preferences and behavior as well as to the effects of different yeast-based diets on SWD flies. As a first approach, the overall profile of the lipid compounds that were annotated was considered. In this case, differences and similarities found between yeast species did not apparently match with SWD preferences or development. For instance, similarities in the global lipid profile between *M. pulcherrima* and *Candida* sp. were observed (Figure 2A). In previous studies [6], these two species had different effects on the survival of SWD larvae: the two species *H. uvarum* and *Candida* sp. had a beneficial effect on the survival of SWD larvae compared to larvae reared on *M. pulcherrima*. The yeast *H. uvarum* has been found to be more attractive for SWD flies compared to other *Drosophila*-associated yeasts, including *S. cerevisiae* and *I. terricola*, when offered in a choice test [19]. In addition, Lewis and Hamby [7] demonstrated that larvae of SWD prefer to feed on *H. uvarum* compared to *S. cerevisiae* and *I. terricola*, while Spitaler et al. [52] showed that SWD adults benefit from *H. uvarum* and *S. vini* in their diet compared to other species, including *S. cerevisiae*. In this study, *H. uvarum* and *S. cerevisiae* were found to have a similar global lipid profile. Considering the lipid profile in its entirety can be misleading. The fact that SWD flies or larvae do not respond similarly to yeasts that have a generally similar lipid profile does not necessarily indicate that lipids do not have an influence on SWD. Rather, specific compounds or compound classes may play a more relevant role in this mechanism. The information concerning specific compounds is hidden though behind global similarities and diversities. To overcome this problem, another approach was applied for the comparison of the two species *H. uvarum* and *S. cerevisiae*. These two yeasts were chosen because, although their lipid profiles were similar, previous studies showed that they give different responses in association with SWD [19]. Additionally, S.c. S288c. is a laboratory strain, while *H. uvarum* was isolated from grapes infested by SWD flies and is frequently reported in association with SWD [5,55]. A chemical enrichment analysis, an innovative tool for improving the biological and biochemical interpretation of metabolomic data, was performed. ChemRICH is a statistical analysis based on chemical similarities and diversities between groups of metabolites. This approach is normally used for evaluation of metabolic changes like an increase or reduction of the concentrations of specific compound classes that occur moving from one metabolic condition to another [69]. It may be used for the comparison of healthy and unhealthy subjects or treated and control samples. In this study, it was proposed to compare S.c. S288c with H.u. 3.4. Results of the analysis are visualized in a two-dimensional impact plot representing the significantly altered lipid clusters (Figure 5). Enrichment results for each group are reported in Table S2. Lipids belonging to the classes of saturated TG, unsaturated PC, LPC, unsaturated FA, and unsaturated PI were found to have a lower concentration in H.u. 3.4 compared to S.c. S288c (blue nodes), while a higher number of more concentrated saturated FA and unsaturated PE were found in H.u. 3.4 (red nodes). Unsaturated TG, which include the highest number of significantly impacted compounds, and unsaturated DG are characterized by a similar number of lipids having higher or lower concentrations in either of the two yeasts (purple nodes). This approach highlights and categorizes the classes of compounds responsible for these diversities, rather than evidencing the differences based on the global profile of lipids. In this way, it was possible to underline differences between two species, which appear similar based on their global lipid profile. This allows us to focus on specific groups of metabolites that may be considered for further studies like feeding trials and behavioral or survival assays. Spitaler et al. [52] already suggested a possible relationship between the presence of specific polar metabolites, their concentrations, and the survival and feeding stimulation of SWD adults. In this study, a broad qualitative approach was used including a wide range of non-polar compounds. Considering that the same yeast species and microorganisms were grown and collected under the same conditions reported in the paper by Spitaler et al. [52], the results of this work represent an implementation of the above-mentioned study and may support future entomological studies.

## 3. Materials and Methods

### 3.1. Chemicals and Growth Media

Formic acid (LC-MS grade) and MTBE (methyl tertiary butyl ether) (HPLC grade) were obtained from Merck KGaA (Darmstadt, Germany). Acetonitrile (LC-MS grade), isopropanol (LC-MS grade) and methanol (LC-MS grade) were purchased from VWR International Srl (Milan, Italy). Toluene (HPLC grade), ammonium formate (LC-MS grade), ammonium acetate (≥98%), and analytical internal standards were purchased from Sigma Aldrich (St. Louis, MO, USA). Potato Dextrose Broth (PDB) and Potato Dextrose Agar (PDA) used for yeast cultivation were obtained from Becton, Dickinson and Company (Sparks, MD, USA).

### 3.2. Yeasts Cultivation and Lipid Extraction

Seven yeast cultures with strain numbers containing LB-NB were isolated from feeding channels of SWD larvae in infested grapes in South Tyrol in 2009 [6]. *S. cerevisiae* strain S288c is a laboratory strain. Yeasts were grown in 220 mL PDB at 25 °C for 30 h in a 250 mL Erlenmeyer flask closed with cotton and aluminum foil on a rotary shaker at 120 rpm. Based on preliminary trials after 30 h, all yeast cultures reached the stationary growth phase. The inoculum (0.1 mL) was prepared with a loop full of yeast cells cultivated on PDA for four days, which were transferred in a 2-mL Eppendorf tube filled with 1 mL PDB and vortexed for 10 s at 1800 rpm. Six replicates of the inoculum were prepared for each yeast. The growth of yeasts was evaluated by measuring the optical density at 600 nm (OD_600_) (Cary 60 UV-Vis, Agilent) and the yeast cell dry weight (CDW) after 30 h (centrifugation of fermentation broth, removal of the supernatant, freeze-drying). The list of yeasts included in the study and values of OD_600_ and CDW are shown in Table 1.

For the extraction of intracellular lipids, 10 mL of fermentation broth were quenched in 20 mL methanol at −80 °C and centrifuged (Eppendorf Centrifuge 5810 R) at −10 °C for 5 min at 4000 rpm using a −80 °C prechilled rotor. The supernatant was discarded, and the pellet was freeze-dried. Ten mg of freeze-dried cell pellet was weighted, and intracellular lipids were extracted using the procedure of Showalter et al. [70]. A volume of 225 µL of −20 °C methanol containing an internal standard mixture of PE (17:0/17:0), PG (17:0/17:0), sphingosine (d17:1), ceramide (d18:1/17:0), SM (d18:0/17:0), FA (16:0)-d3, PC (12:0/13:0), cholesterol d7, TG d5 (17:0/17:1/17:0), DG (12:0/12:0/0:0), DG (18:1/2:0/0:0), MG (17:0/0:0/0:0), LPC (17:0), LPE (17:1) and 750 μL of −20 °C MTBE containing the internal standard cholesteryl ester 22:1 was added to the pellet. Samples were shaken for 6 min at 4 °C using a Thermomixer (Eppendorf) and 188 μL of milliQ water was added. Samples were vortexed, centrifuged, and 350 µL of the upper layer was collected, evaporated to dryness using a SpeedVac vacuum concentrator, and re-suspended in methanol:toluene (9:1, *v*/*v*) containing 50 ng mL^−1^ CUDA ((12-[[(cyclohexylamino)carbonyl]amino]-dodecanoic acid). Samples were vortexed, sonicated for 5 min, and centrifuged before analysis. Pooled samples were used as a quality control.

### 3.3. Chromatographic and Mass Spectrometric Conditions

Chromatographic conditions were based on Showalter et al. [70]. A Waters Acquity UPLC CSH C18 (100 mm length × 2.1 mm id; 1.7 μm particle size) column with a Waters Acquity VanGuard CSH C18 pre-column (5 mm × 2.1 mm id; 1.7 μm particle size) maintained at 65 °C was used for RPLC-QTOFMS analysis. In positive ion mode, solvent A was 60:40 *v*/*v* acetonitrile:water with 10 mM ammonium formate and 0.1% formic acid and solvent B was 90:10 *v*/*v* isopropanol:acetonitrile with 10 mM ammonium formate and 0.1% formic acid. In negative ion mode, solvent A was 60:40 *v*/*v* acetonitrile:water with 10 mM ammonium acetate and solvent B was 90:10 *v*/*v* isopropanol:acetonitrile with 10 mM ammonium acetate. The flow rate was set at 0.6 mL/min, with a 15 min gradient as reported in the paper.

The instrument Impact HD QTOF (Bruker) equipped with an Ultimate 3000 UHPLC (Thermo Scientific, Waltham, MA, USA) was used for LC-MS analysis. Five µL of the re-suspended sample was injected in ESI positive ion mode, while the injection volume in negative ion mode was 10 µL. The mass spectrometric conditions were as follows: *m/z* range, 60–1700; capillary voltage, 3500 V; nebulizer gas (nitrogen), 2.4 bar; dry gas (nitrogen), 8 L/min in positive ion mode and 12 L/min in negative ion mode; dry temperature, 325 °C in positive ion mode and 200 °C in negative ion mode. For MS/MS, the collision energy was set at 20 eV in positive and in negative ion mode, and the spectra rate was 13 Hz with 4 precursor ions per cycle. Sodium formate was used as a calibrant for maintaining mass accuracy.

### 3.4. Data Processing and Statistics

For compound identification, full-scan and MS/MS analyses were performed. MS-DIAL was used for deconvolution, peak picking, alignment, and annotation [71]. LipidBlast was used as the library for compound identification with an identification score cut-off of 85% and a retention time tolerance of 0.1 min. The level of identification of lipids was two for all compounds based on Sumner et al. [72]. The nomenclature used was based on Züllig et al. [73]: the bond type level was reported in the case of annotation based on a high-resolution full-scan (MS1), while double-bond positions were indicated in case of MS/MS-based annotation (Table S1). Peak heights were submitted to Metaboanalyst. Values were normalized using class-based internal standards and further by log transformation before statistical testing. To assess the quality of the data, pooled quality control samples (QCs) were distributed evenly in the analytical batches, RSD% among the QCs of each internal standard was calculated (Table S3), and the clustering of QCs samples was visually inspected through PCA (Table S3). Pareto scaling was performed for PCA. ANOVA with Tuckey HSD post hoc testing, Student *t*-tests, and pairwise MANOVA tests were done in SPSS (IBM SPSS statistic 24). Graphs were generated using R [74]. To evaluate significantly impacted lipid clusters between S.c. S288c and H.u. 3.4, a chemical similarity enrichment analysis (ChemRICH) was performed using the Kolmogorov–Smirnov-test for statistical analysis [69].

Lipidomics data have been deposited into the EMBL-EBI MetaboLights database [75] with the identifier MTBLS1955. The complete dataset can be accessed here: https://www.ebi.ac.uk/metabolights/MTBLS1955.

## 4. Conclusions

The yeast strain has an effect on the attractiveness to SWD flies and affects SWD feeding preferences. Lipids constitute a nutritional source for *Drosophila* flies; some of these molecules are detected by their gustatory system and are precursors of aromatic compounds. In this study, an untargeted explorative approach was used to investigate the differences in the lipid profile among yeast species naturally occurring in association with SWD. The results constitute a starting point for future investigations on the effects of specific chemical compounds on the behavior of SWD flies. Differences between species were highlighted, as well as the classes of compounds mostly responsible for the discrimination between yeasts. A number of 171 metabolites were annotated. Three strains of *H. uvarum* under investigation were found to be very similar, and all the other species could be distinguished from each other based on their lipid profiles. ChemRICH enrichment analysis was performed between a laboratory strain (*S. cerevisiae*) and a species frequently found in association with SWD flies (*H. uvarum*) to point out diversities within the lipid classes between two species that were reported to differently affect SWD behavior. Significant differences in clusters of lipids were found between the two yeast species. Compounds with significantly higher or lower amounts in either of the two species under investigation belonged to the lipid classes TG, DG, FA, and GP. The information about the differences in the lipid profiles of yeast species associated with SWD may be useful for further entomological and behavioral studies concerning the complex interaction between specific yeasts and insects.

## Figures and Tables

**Table 1:** A list of yeasts included in the dataset. Values (Mean ± SD) of optical density at 600 nm (OD_600_) and cell dry weight (CDW) of the fermentation broths (n = 6) are reported.

Yeast Species	Strain	Accession Number *	Abbreviation	OD_600_	CDW(mg mL^−1^ Fermentation Broth)
*Saccharomyces cerevisiae*	S288c	-	S.c. S288c	1.98 ± 0.03	1.57 ± 0.18
*Hanseniaspora uvarum*	LB-NB-1.21	KP298009	H.u. 1.21	1.86 ± 0.02	1.42 ± 0.11
*Hanseniaspora uvarum*	LB-NB-2.2	MK567898	H.u. 2.2	1.83 ± 0.05	1.48 ± 0.15
*Hanseniaspora uvarum*	LB-NB-3.4	MK567905	H.u. 3.4	1.86 ± 0.04	1.59 ± 0.06
*Issatchenkia/Picchia terricola*	LB-NB-2.1	MK567903	I.t. 2.1	1.90 ± 0.04	1.32 ± 0.17
*Metschnikowia pulcherrima*	LB-NB-3.2	KP298012	M.p. 3.2	2.02 ± 0.05	1.94 ± 0.15
*Saccharomycopsis vini*	LB-NB-1.33	KP298011	S.v. 1.33	1.78 ± 0.10	1.63 ± 0.13
*Candida* sp.	LB-NB-3.3	KP298013	C.sp. 3.3	2.04 ± 0.07	1.77 ± 0.25

* The accession numbers were deposited in the GenBank NCBI.

## Supplementary Materials

The following are available online at https://www.mdpi.com/2218-1989/10/9/352/s1, Table S1: List of the compounds annotated in the yeasts included in the study (mean ± SD, n = 6). Peak height values were normalized using class-based internal standards. The internal standards used for the normalization of the peak heights of each compound are reported. The level of identification of lipids for all compounds was two based on Sumner et al. (2007; doi:10.1007/s11306-007-0082-2). Table S2: Results of the ChemRICH analysis of H.u. 3.4 versus S.c. S288c., Table S3: List of the internal standards used. Peak heights found in quality control pooled samples (QCs) and relative standard deviation (RSD%) are reported for each internal standard. Two-dimensional score plots of all samples, including QCs, generated with all the annotated compounds using the first two principal components of the PCA are reported for the raw dataset and after class-based internal standard normalization and log transformation.
